# Impact of guideline awareness in public pharmacies on counseling of patients with acute or chronic constipation in a survey of pharmacy personnel

**DOI:** 10.1186/s12876-020-01338-4

**Published:** 2020-06-17

**Authors:** Marion Eberlin, Sabine Landes, Doerthe Biber-Feiter, Martin C. Michel

**Affiliations:** 1 Consumer Healthcare Medical Affairs, Sanofi-Aventis Deutschland GmbH, Industriepark Hoechst, 65026 Frankfurt am Main, Germany; 2 Consumer Healthcare CMI, Sanofi-Aventis Deutschland GmbH, Industriepark Hoechst, Frankfurt am Main, 65026 Germany; 3 Department of Pharmacology, Johannes Gutenberg University, Mainz, Germany

**Keywords:** Constipation, Self-medication, Treatment guideline

## Abstract

**Background:**

Constipation is often self-managed by patients and guidelines are available to aid healthcare professionals in the counseling of patients for self-management. Therefore, we have explored the knowledge and attitude of pharmacy personnel towards guidelines for the management of acute and functional chronic constipation and how they affects their recommendations.

**Methods:**

An online survey was conducted among 201 pharmacists and pharmacy technicians from an existing panel. They were presented with two typical cases, a 62-year old woman with functional chronic constipation and a 42-year old woman with travel plans. For each case, they were asked about their treatment recommendations and the underlying rationale. Thereafter, they were provided with contents from an applicable national guideline and asked again about their recommendations and the underlying rationale. In line with the exploratory nature, data were analyzed in a descriptive manner only.

**Results:**

Before exposure to guideline content, the most frequent recommendations for chronic constipation were macrogol, fiber and lactulose and for acute constipation sodium picosulfate, bisacodyl and enemas. Following guideline exposure, the most frequent recommendations for chronic constipation were macrogol, bisacodyl and sodium picosulfate and for acute constipation bisacodyl, sodium picosulfate and macrogol (all three equally recommended by the guideline for the management of acute and chronic constipation). Correspondingly, the rationale behind the recommendations shifted with guideline conformity becoming a leading reason.

**Conclusions:**

Awareness of the content of an applicable guideline on the management of constipation was poor among pharmacy personnel. Accordingly, recommendations in many cases were not in line with the guideline. Greater awareness of guideline content is desirable to enable more evidence-based recommendations in the management of constipation.

## Background

Constipation is a prevalent condition; specific estimates depend on whether presence verified by the Rome IV criteria [1] or patient-reported incidence are considered; reported estimates range from 2 to 28% [2] and mean prevalence in Europe is estimated at about 15% [3]. The prevalence is increased in women, the elderly and those with low socio-economic status [4–6]. It can be grouped into chronic and occasional/acute constipation. Chronic constipation can occur secondary to neurological (e.g., stroke, Parkinson’s disease) and metabolic diseases (e.g., diabetes, hypothyroidism), intestinal surgery or medications (e.g., opioids or muscarinic antagonists) [7]. However, no primary cause is identified in many cases, which are summarized as “functional constipation”. Chronic constipation has a major adverse impact on quality of life [8] and represents an economic burden to patients and healthcare providers, largely due to resource utilization [9]. Much less is known about the prevalence of occasional/acute constipation, at least partly because there is no general definition of the condition.

Constipation is often managed successfully by self-medication with prescription-free medicines [10]. This represents an acceptable approach if certain conditions are met. In this regard, professional societies in Germany in association with patient organizations have developed a guideline for healthcare professionals (HCPs) and sufferers including recommendations for patients on the use of self-medication with laxatives [3]. Step 1 in the self-management of constipation should consider general measures such as lifestyle changes or ingestion of more fiber (dietary fiber/fiber supplements). Step 2 recommends the use of medications such as bisacodyl, macrogol and sodium picosulfate (SPS), which are available without a prescription in Germany. All three medications are recommended equally as first-line treatment options for acute and chronic constipation, and the choice among them should be driven by patient preference according to the guideline.

Guidelines exist in many areas of medicine but awareness of and adherence to them by HCPs is not always optimal. Some previous studies have explored adherence to guidelines on the management of constipation in children in various regions of the world [5, 11–15]. They consistently report a moderate degree of knowledge and adherence to such guidelines. However, we did not identify any such study related to constipation management of adults. Moreover, the above studies investigated guideline knowledge and adherence by physicians and did not include that by other HCPs such as pharmacists (PHs) and pharmacy technicians (PTs). As PHs and PTs provide a major share of patient counseling on the adequate and safe use of laxatives in self-medication, the present study was designed to explore their knowledge and attitude towards guideline recommendations. For the latter, we explored whether providing content from the applicable guideline [3] would change recommendation behavior.

## Methods

We have conducted an online survey in July 2017 on the DocCheck Research platform (www.research.doccheck.com) using an existing panel of German HCPs. Based on the anonymous character of the survey, ethical committee approval was neither required nor recommended by applicable laws and regulations in Germany at the time the survey was performed. As participants were recruited from an existing panel of HCPs having indicated their willingness to contribute to surveys like this, additional participant consent was not required. The survey was planned to include about 200 HCPs working in public pharmacies with about equal representation of PHs and PTs (actual participation 104 PHs and 97 PTs).

The survey first asked whether participants had ever done a dedicated search for treatment recommendations for acute and chronic constipation (yes/no). If yes, a follow-up question asked which sources were used routinely to obtain information on constipation (seven options plus “other, to be specified”; multiple nominations possible). Thereafter, the survey presented two hypothetical cases typical for a pharmacy setting. One was a 62-year old woman with chronic constipation, diagnosed by a physician as chronic functional constipation. The other was a 42-year old woman planning a vacation trip and knowing from previous trips that she often suffers from acute constipation during such trips; she now wished to prophylactically buy a medicine for acute constipation to be used if needed. The verbatim German text of the case descriptions and an English translation are shown in the Online Supplement. Following each case presentation, participants were requested to rank choices for recommendation from the typical portfolio available in Germany, representing the ten most often recommended treatment options (bisacodyl, SPS, macrogol, lactulose, anthrachinones (e.g. extracts from Senna leaves or fruits), salinic laxatives (e.g. MgSO_4_ or Na_2_SO_4_), glycerin, enema, bulk-forming agents and “others” (to be identified if selected)). Options were presented in random order and participants were asked to perform ranking by on-screen drag and drop. An open question asked for the rationale behind their top-three recommendations. The next two questions asked to rank the ten options for efficacy and tolerability, respectively, again with a request to provide a rationale for the top-three choices. A separate question asked to rank the ten options about strength of evidence and underlying reasons (for the top 3 choices). A final question inquired whether the addition of electrolytes was required in macrogol preparations in the treatment of constipation. Thereafter, a summary excerpt of medical treatment recommendations from the applicable German guideline was provided (verbatim text and English translation in Online Supplement). Thereafter, each of the original cases and questions was asked again to explore a possible change of recommendations and underlying reasons.

Data are shown as % of responders. In line with the exploratory nature of the survey, hypothesis-testing statistical analysis has not been performed [16].

## Results

### General information

The majority (77%) of HCPs reported to have done a dedicated search for information on the topic of constipation in the past. Within this group, used sources of information were professional journals (88%), internet (73%), summary of product characteristics (66%), continuing education events from the chamber of pharmacists or of physicians (56%), textbooks (45%), trainings and information offered by sales representatives (38%), conversations with customers (26%), professional societies (8%) and “other” (3%; multiple nominations possible).

In a self-assessment of knowledge on the content of the applicable constipation guidelines, 30% considered their knowledge to be good or very good, 41% to be moderate, and 28% to be limited or poor. After presentation of an excerpt from the treatment guideline [3] (see Online Supplement), the self-assessment of pre-existing awareness shifted with full knowledge being reported by 6%, partial by 64% and lack of knowledge by 29%.

### Pre-guideline recommendations

**Fig. 1:**
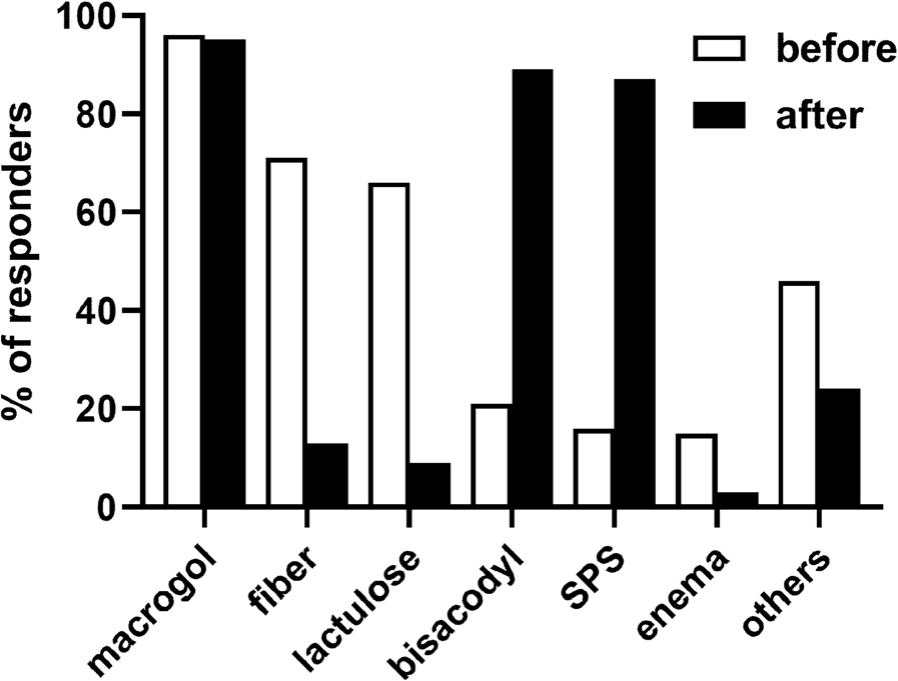
Three most frequently recommended treatments for functional chronic constipation prior to (open bars) and after (filled bars) information on applicable guideline. Selections given by < 5% are not shown

**Fig. 2:**
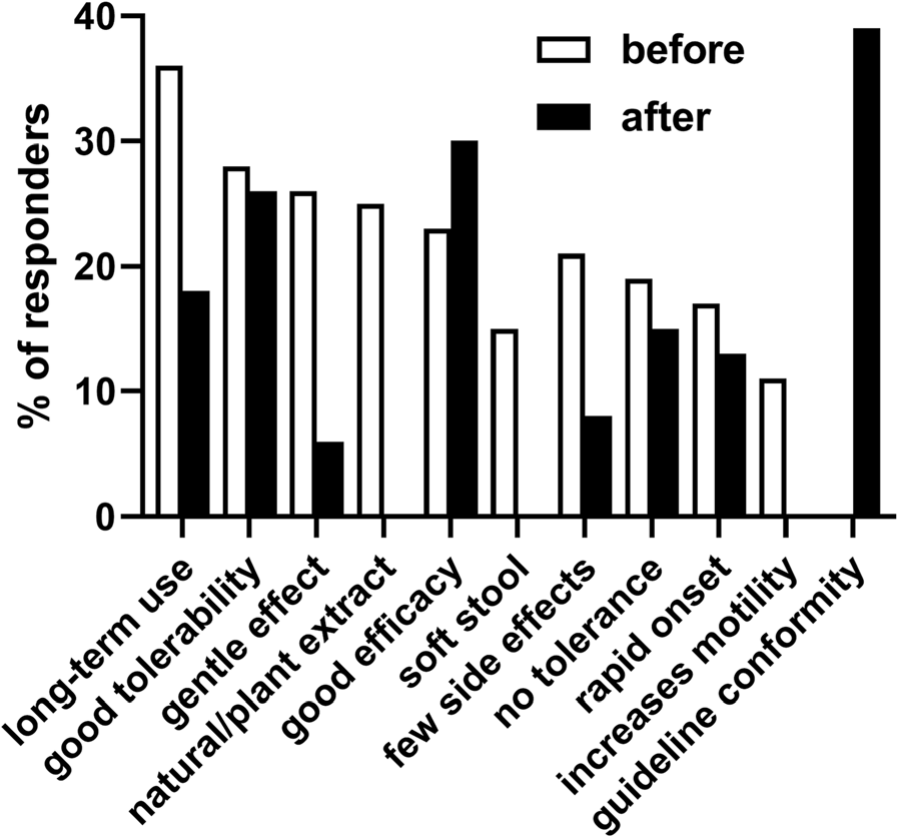
Reasons for selection of recommended treatments for functional chronic constipation. Reasons provided by < 10% are not shown

Prior to presentation of guideline content [3], the most frequently recommended treatment options as part of the top-3 choices for the patient with chronic functional constipation were macrogol (96%), fiber (71%), lactulose (66%) and bisacodyl (21%) with all other options being recommended by < 20% of the HCPs; Fig. 1). The most frequently stated reasons for the preferred recommendations were long-term use (36%), good tolerability (28%), gentle effect (26%), “natural”/plant extract (25%), good efficacy (23%), “softens stool” (22%), “few side effects” (21%) were named most often; several others were also named but by < 20% of HCPs (multiple nominations possible; Fig. 2).

**Fig. 3:**
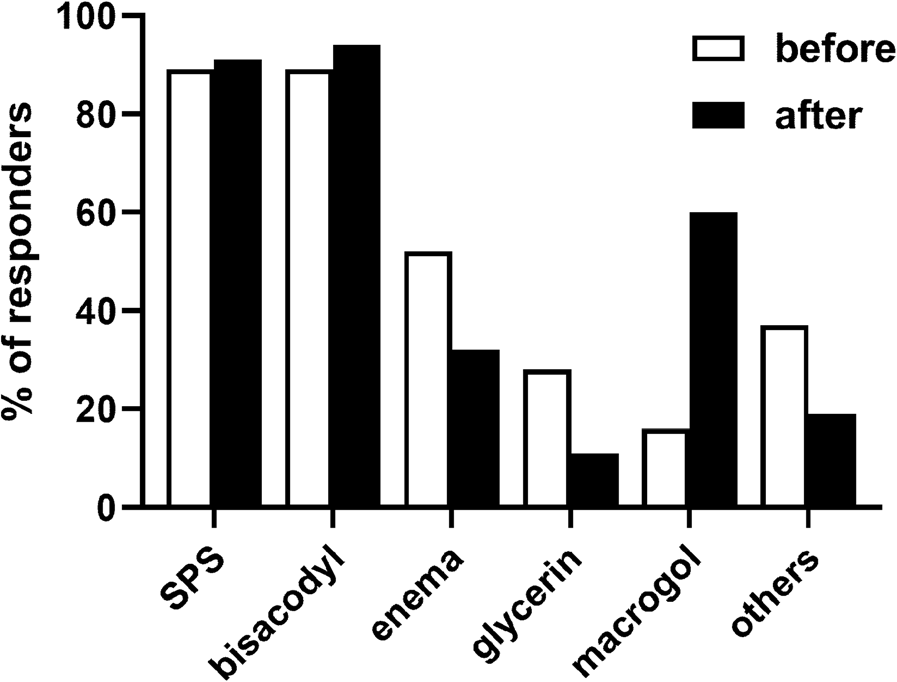
Three most frequently recommended treatments for acute constipation prior to (open bars) and after (filled bars) information on applicable guideline. Selections given by < 6% are not shown

**Fig. 4:**
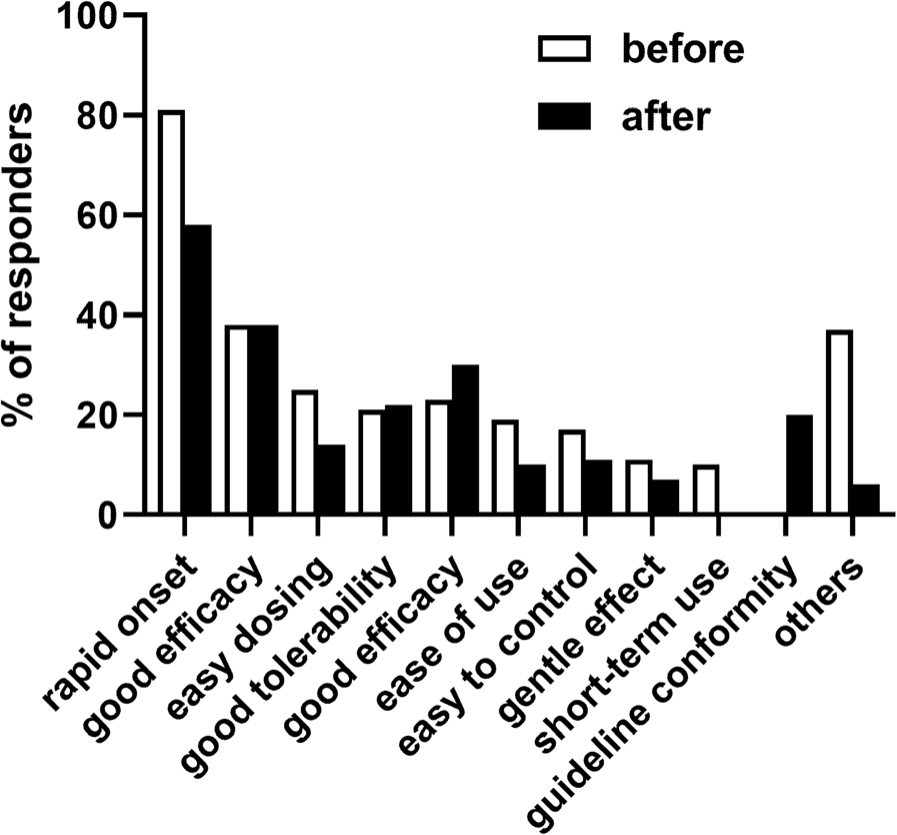
Reasons for selection of recommended treatments for acute constipation. Reasons provided by < 10% are not shown

The most frequently recommended treatment options as part of the top-3 choices for acute constipation were SPS (89%), bisacodyl (89%), enemas (52%) and glycerin (28%) with all other options recommended by < 20% of the HCPs (Fig. 3). The most frequently stated reasons for the recommendations were rapid onset (81%), good efficacy (38%), ease of individual dose-selection (25%) and good tolerability (21%), with several other options named by < 20% of participants (Fig. 4).

### Post-guideline recommendations

After having been shown excerpts from the applicable guideline [3], recommendations changed markedly: While macrogol (95%) remained strong, percentage of bisacodyl (89%) and SPS recommenders increased markedly (87%), whereas that of fiber (13%) and lactulose (9%) decreased markedly (Fig. 1). Thus, recommendations for bisacodyl and SPS increased by 68% point and 70% points, respectively, whereas that for fiber and lactulose decreased by 58% points and 56% points, respectively. This shift in recommendation was accompanied by a shift of underlying reasons: the most frequently stated reasons were guideline conformity (39%), good efficacy (30%) and good tolerability (26%) with all other reasons being stated by < 20% of participants.

Showing of the applicable guideline [3] also affected recommendations for treatment options of acute constipation: While bisacodyl (94%) and SPS (91%) remained strong, recommendations for macrogol increased (60%), whereas those for enemas (32%) and glycerin (11%) decreased (Fig. 3). Thus, recommendation of macrogol increased by 43% points and that for enemas and glycerin decreased by 20 and 17% points, respectively. In contrast to the major shift in reasons for the recommendations in chronic constipation (see above), reasons for the recommendations in acute constipation changed only to a limited extent (Fig. 4). Thus, rapid onset of effect (58%), good efficacy (38%) and good tolerability (22%) remained in the group of most frequently named reasons; however, guideline conformity (20%) became a stronger and ease of individual dosing (14%) a weaker reason for recommendation.

## Discussion

Chronic and acute (occasional) constipation are prevalent conditions [2, 3] that adversely affect the wellbeing of affected subjects and have considerable socio-economic impact. A sizeable fraction of patients report constipation that does not meet the Rome criteria [17], a phenomenon named No Rome Constipation [18]. Many patients with self-reported constipation do not seek advice by a physician [17] and patients with chronic constipation who are in professional care are often unsatisfied with the treatments they have been offered [19]. When certain causes have been excluded and a functional constipation has been established, self-management of patients by lifestyle modification as well as medicinal products are considered recommended treatment steps according to guidelines [3]. This is reflected in physician behavior in primary care [20]. The (self-)management of constipation often involves prescription-free medications obtained from pharmacies and/or drug stores (depending on applicable local legislation). The concept and regulation of self-medication differs between countries. In Germany, pharmacy personnel is obliged to offer counselling on choice of non-prescription medication, but the patient is free to make her/his own choice including refusing to listen to counseling or to purchase a prescription-free medication against advice by the pharmacist.

As multiple options are available for the medical treatment of constipation [21, 22], HCPs working in pharmacies have a key role in advising patients on the appropriate use and selection of laxatives. Studies among physicians treating constipation in children have consistently reported limited knowledge of and adherence to applicable guidelines [5, 11–15]. The state of knowledge of PHs and PTs advising patients with constipation is less clear. Therefore, we have performed an online survey using an existing panel of HCPs based in public pharmacies to explore their knowledge and attitudes towards the self-treatment options of constipation.

Most (77%) of HCPs in our survey reported to have done dedicated searches for information on constipation and having used a variety of resources for such searches. Sources of information independent of the pharmaceutical industry were among the most often used. While 30 and 41% of participating HCPs felt to have a good to very good or at least a moderate knowledge on the content of the applicable guidelines in Germany [3], most of them realized after presentation of excerpts from the guideline that they had been over-confident in this regard.

To explore knowledge and attitude of HCPs, we presented them with two typical cases, one suffering from functional chronic constipation and one wishing to preventively obtain a product for the management of acute constipation (Online Supplement). For each clinical case, we asked them to rank frequently used prescription-free treatments and explain their reasoning behind those choices. Thereafter, HCPs were presented with excerpts of the applicable guideline [3] and then were asked again to rank available prescription-free treatments and explain the reasoning behind their choices. Interestingly, choices and underlying reasoning differed considerably between the case with functional chronic and with anticipated acute constipation.

The applicable guideline for chronic functional constipation [3] provides three first-line options if general measures have yielded insufficient efficacy: bisacodyl, macrogol and SPS. However, HCPs endorsed bisacodyl and SPS much less frequently than the guideline would recommends. In contrast, fiber and lactulose were recommended much more frequently than bisacodyl or SPS by participating HCPs. However, most patients with chronic functional constipation have already tried managing their condition by increased fiber intake – and failed. Moreover, lactulose is not recommended as first-line treatment in the guideline. Correspondingly, reasons such as intended long-term use, perceived gentle effect and being a “natural”/phytotherapeutic remedy featured highly among stated reasons despite not being listed as major rationale for the guideline-recommended treatments. Studies showing good efficacy and tolerability along with high patient satisfaction with bisacodyl or SPS [23, 24] were also apparently not a major consideration in recommendations. Following presentation of excerpts from the guideline, recommendations shifted (more frequent recommendation of bisacodyl and SPS, less frequent of bulking agents and lactulose), which is much more in line with the guideline [3]. Conversely, the use of macrogol was recommended rarely for the case with acute constipation, but that recommendation surged after presentation of guideline excerpts. Of note, the existence of a guideline recommendation was not provided as a reason for recommendation before excerpts from the guideline had been presented but became a frequently named reason thereafter.

## Conclusions

We conclude that awareness of the content of an applicable guideline [3] is poor among PHs and PTs working in public pharmacies in Germany. This is in line with previous studies of physicians treating constipation in children [5, 11–15]. While it is likely that this similarly applies to physicians involved in the care of adult with constipation, this needs to be determined in future studies. Accordingly, recommendations based on fictional but typical case studies differed considerably from those in the guidelines. However, HCPs rapidly changed their recommendations after having been exposed to excerpts from the guideline. Taken together these data suggest that guideline awareness is poor but better awareness leads to immediate changes and more evidenced-based recommendations, potentially resulting in a better management of symptoms. To this end, mobile apps on guideline content could be developed or integration of links to such information could be embedded in the software packages used by pharmacists and physicians managing their patients. While we assume that limited guideline awareness and behavioral changes upon exposure to applicable guidelines will change recommendations in other therapeutic areas as well, this remains to be explored. Moreover, it needs to be studied whether acute exposure to guideline content will lead to long-lasting changes in recommendations.

## Supplementary information

**Additional file 1.**

## Data Availability

The datasets generated and/or analyzed during the study are available from the corresponding author upon reasonable request.

## Abbreviations

HCP: Healthcare professional
PT: Certified pharmacy technician
PH: Pharmacist
SPS: Sodium picosulfate
