# Supplementary material for: Impact of guideline awareness in public pharmacies on counseling of patients with acute or chronic constipation in a survey of pharmacy personnel
Source: BMC Gastroenterol. 2020 Jun 17;20:191. doi: 10.1186/s12876-020-01338-4 (PMC7301513; doi:10.1186/s12876-020-01338-4)
Supplement: Supplementary file 1 — Additional file 1. [file 12876_2020_1338_MOESM1_ESM.docx]

Online supplement to

**Impact of guideline awareness in public pharmacies on counseling of patients with acute or chronic constipation in a survey of pharmacy personnel**

Marion Eberlin, Sabine Landes, Doerthe Biber-Feiter, Martin C. Michel

Case 1 original German text:

Eine 62-jährige Patientin klagt seit Jahren über bestehenden, klumpigen, harten Stuhl, häufigen Stuhldrang und Verstopfung. Weiterhin gibt sie an, ständig Unterbauchschmerzen, Völlegefühl und Blähungen zu haben. Sie berichtet, dass sie die Verstopfungsursache bereits ärztlich abgeklärt hat, sich eine konkrete Verstopfungsursache jedoch nicht finden ließ. Die ärztliche Diagnose lautet funktionelle chronische Verstopfung. Diese Patientin kommt regelmäßig in Ihre Apotheke und sucht Rat.

Case 1 (chronic constipation, returning patient):

A 62-year old female patient has been complaining for years about lumpy, hard stools, frequent urge to defecate and constipation. Moreover, she states to have constantly lower abdominal pain and bloating. She reports that the cause of her constipation has already been evaluated by a physician, but no specific cause had been identified. The medical diagnosis was functional chronic constipation. The patient visits the pharmacy regularly and asks for advice.

Case 2 original German text:

Eine 42-jährige Patientin möchte in einer Woche in den Urlaub nach Frankreich fahren. Aus Erfahrung weiß sie, dass sie auf Reisen oft unter einer akuten Verstopfung leidet. Für ihre Reiseapotheke möchte sie daher etwas kaufen, um im Fall der Fälle gut ausgerüstet zu sein. Sie sucht hierfür Ihren Rat.

Case 2 (acute constipation):

A 42-year old female patient plans to go on vacation in France a week from now. Based on prior experience, she knows that she frequently suffers from acute constipation while travelling. She wishes to purchase something for her travel pharmacy to be prepared if that occurs. She asks for advice.

Excerpt of German-language guideline for the treatment of constipation (Andresen et al., 2011) original German text:

- Chronische Verstopfung ist keine banale Befindlichkeitsstörung, welche durch falsche Lebensgewohnheiten selbst verschuldet und damit leicht zu korrigieren ist, sondern ein **Symptom mit Krankheitswert** (zahlreiche unangenehme Symptome und deutlich eingeschränkte **Lebensqualität**). Die unbehandelte chronische Verstopfung kann Risiken birgen (z.B. Darmverschluss, Mikrobiomveränderungen und Folgen, kardiovaskuläre Risiken etc.).

- Eine rechtzeitige Therapie mit wirksamen, verträglichen und sicheren Arzneimitteln ist notwendig, um die Lebensqualität zu verbessern und um ggf. Komplikationen und Risiken der Verstopfung zu reduzieren.

- Die aktuelle deutsche Leitlinie empfiehlt Bisacodyl, Natriumpicosulfat und Macrogol gleichwertig als **Mittel der 1. Wahl** in der medikamentösen Therapie **chronischer Verstopfung** und auch **akut funktioneller Verstopfung**. Allgemeinmaßnahmen, wie die **Normalisierung** der Trinkmenge, der Bewegung und der Ballaststoffzufuhr sollen versucht werden. Wenn diese Maßnahmen nicht ausreichen und/oder schlecht vertragen werden, wird die Einnahme der Mittel der 1. Wahl empfohlen.

- Wenn die Verstopfungsursache ärztlich abgeklärt ist, können Patienten diese Wirkstoffe **langfristig** einnehmen. Es kommt dann **weder zu Gewöhnung (kein Wirkverlust, keine Dosissteigerungen), noch zu Elektrolytverlusten**. Wichtig ist der **bestimmungsgemäße Gebrauch** = Dosierung für einen **weichen, geformten Stuhl** (kein anhaltender Durchfall).

- Die Einnahme aller drei Wirkstoffe ist auch **in der Stillzeit möglich**.

- Bisacodyl und Natriumpicosulfat erhöhen nicht nur deutlich die Darmbewegung, sondern **verbessern auch signifikant die Lebensqualität** der Patienten.

- Bei der Verwendung von Macrogolen als Abführmittel bei Verstopfung ist **die Zugabe von Elektrolyten nicht notwendig**, da bei bestimmungsgemäßer Anwendung keine Elektrolyte verloren gehen (nur bei Darmlavage oder Therapie der Koprostase erforderlich). Elektrolytfreie Präparate schmecken außerdem weniger schlecht.

Excerpt of German-language guideline for the treatment of constipation (Andresen et al., 2011) English translation:

- Chronic constipation is not a banal mood disorder that is caused by wrong lifestyle and, accordingly, can easily be corrected; rather, it is a **symptom with clinical significance** (many unpleasant symptoms and markedly reduced **quality of life**). Untreated chronic constipation can harbor risks (e.g. bowel obstruction, changes of microbiome and consequences, cardiovascular risks etc.).

- A timely treatment with effective, well tolerated and safe medicines is required to improve quality of life and, if applicable, reduce complications and risks of constipation.

- The current German guideline recommends bisacodyl, sodium picosofulate and macrogol as equally ranked **first choices** in the medical treatment of **chronic constipation** and also of **acute functional constipation**. General measures such as **normalization** of fluid intake, physical activity and dietary fiber shall be tried. When these measures are insufficient and/or poorly tolerated, ingestion of a medicine of first choice is recommended.

- If the cause of constipation has been evaluated by a physician, patients can use these medicines **long-term**. There is **neither habituation (no loss of effect, no dose escalation), nor loss of electrolytes**. Correct use = dosing for a soft, formed stool (no sustained diarrhea) is important.

- All three medicines can also be used during the lactation period.

- Bisacodyl and sodium picosulfate not only increase gut movements markedly, but also **significantly improve quality of life** of patients.

- The **addition of electrolyte is not required** in the use of macrogol as laxative in constipation, because no electrolytes are lost upon intended use (only required during gut lavage or treatment of coprostasis). Electrolyte-free preparations taste better.

REFERENCE

Andresen V, Enck P, Frieling T, Herold A, Ilgenstein P, Jesse N, Karaus M, Kasparek M, Keller J, Kuhlbusch-Zicklam, R., Krammer H, Kreiss M, Layer P, Madisch A, Matthes H, Mönnikes H, Müller-Lissner S, Preiss J, Sailer M, Schemann M, Schwille-Kiuntke J, Voderholzer W, van der Voort I, Wedel T and Pehl C (2011) S2k-Leitlinie chronische Obstipation: Definition, Pathophysiologie, Diagnostik und Therapie. *Z Gastroenterol* **51**:651-672.
